# Supplementary material for: Novel Isoform DTX3c Associates with UBE2N-UBA1 and Cdc48/p97 as Part of the EphB4 Degradation Complex Regulated by the Autocrine IGF-II/IRA Signal in Malignant Mesothelioma
Source: Int J Mol Sci. 2023 Apr 17;24(8):7380. doi: 10.3390/ijms24087380 (PMC10139083; doi:10.3390/ijms24087380)
Supplement: Supplementary file 1 [file ijms-24-07380-s001.zip › ijms-2268578-supplementary.pdf]

***Supplemental Table S1**. Antibodies and related reagents used for the study*				
Antigen (/Ab Clone)	*Animal source/ clonality*	*Working concentration*	*Provider*	*Clone /product#*
α-IGF-II neutralizing mAb	goat monoclonal	1.15 μg/ml	R&D Systems	AF292
α-EphB4 (H-10)	mouse monoclonal	1:2000	Santa Cruz	H-10/sc-365510
α-VCP(cdc48)/p97(D9)	mouse monoclonal	1:1000	Santa Cruz	D-9/sc133212
α-VCP(cdc48)/p97	rabbit polyclonal	1:1000	Sigma Aldrich	HPA012728
α-DTX3 (C10)	mouse monoclonal	1:1000	Santa Cruz	C10/sc376439
α-UBA1 (2G2)	mouse monoclonal	1:1000	Santa Cruz	2G2/sc53555
α-UBE2N (4E11)	mouse monoclonal	1:1000	Santa Cruz	4E11/sc58452
α-Ubiquitin (P4D1)	mouse monoclonal	1:1000	Santa Cruz	P4D1/sc8017
Anti-mouse IgG-HRP	chicken monoclonal	1:2000	Santa Cruz	sc2954
Anti-IgGk-LC-HRP	mouse monoclonal	1:1000	Santa Cruz	sc156102
Anti-mouse-IgG-mag beads (Dynabeads)	goat	2 μl/ml	Dynal Biotech	110.33
Streptavidin mag beads	n/a	2 μl/ml	Genscript	L00424
Protein A/G beads	n/a	10 μl/condition	Oncogene	
MagnaChip Protein A/G Magnetic Bead	n/a	5 μl/condition	Millipore	16-663X
Dimethyl-pimelimidate	n/a	20 mM	Sigma Aldrich	D8388
SPDP	n/a	2 mM	Sigma Aldrich	P3415
Ubiquitin, human synthetic	n/a	800 ng/rxn	Boston Biochemical	U-100H
MG132	n/a	200 nM	Calbiochem	474790
ML240	n/a	200 nM	SIGMA	SML1071
